# Tracking Changes in Students’ Online Self-Regulated Learning Behaviors and Achievement Goals Using Trace Clustering and Process Mining

**DOI:** 10.3389/fpsyg.2022.813514

**Published:** 2022-03-17

**Authors:** Michelle Taub, Allison M. Banzon, Tom Zhang, Zhongzhou Chen

**Affiliations:** ^1^Department of Learning Sciences and Educational Research, University of Central Florida, Orlando, FL, United States; ^2^Department of Physics, University of Central Florida, Orlando, FL, United States

**Keywords:** achievement goal orientation, online learning modules, process mining, self-regulated learning, trace clustering

## Abstract

Success in online and blended courses requires engaging in self-regulated learning (SRL), especially for challenging STEM disciplines, such as physics. This involves students planning how they will navigate course assignments and activities, setting goals for completion, monitoring their progress and content understanding, and reflecting on how they completed each assignment. Based on Winne & Hadwin’s COPES model, SRL is a series of events that temporally unfold during learning, impacted by changing internal and external factors, such as goal orientation and content difficulty. Thus, as goal orientation and content difficulty change throughout a course, so might students’ use of SRL processes. This paper studies how students’ SRL behavior and achievement goal orientation change over time in a large (*N* = 250) college introductory level physics course taught online. Students’ achievement goal orientation was measured by repeated administration of the achievement goals questionnaire-revised (AGQ-R). Students’ SRL behavior was measured by analyzing their clickstream event traces interacting with online learning modules *via* a combination of trace clustering and process mining. Event traces were first divided into groups similar in nature using agglomerative clustering, with similarity between traces determined based on a set of derived characteristics most reflective of students’ SRL processes. We then generated causal nets for each cluster of traces *via* process mining and interpreted the underlying behavior and strategy of each causal net according to the COPES SRL framework. We then measured the frequency at which students adopted each causal net and assessed whether the adoption of different causal nets was associated with responses to the AGQ-R. By repeating the analysis for three sets of online learning modules assigned at the beginning, middle, and end of the semester, we examined how the frequency of each causal net changed over time, and how the change correlated with changes to the AGQ-R responses. Results have implications for measuring the temporal nature of SRL during online learning, as well as the factors impacting the use of SRL processes in an online physics course. Results also provide guidance for developing online instructional materials that foster effective SRL for students with different motivational profiles.

## Introduction

Self-regulated learning (SRL) behaviors are an essential component of post-secondary students’ academic success, especially in courses covering complex topics like physics and calculus. Incoming undergraduates often transition from high school into large, blended learning environments that may provide reduced direct instruction and fewer opportunities for students to engage with instructors. These differences require learners to navigate their course work with increased independence, taking a more active role in their own instruction (Schunk and Zimmerman, 2011). Without the external supports traditionally provided in high school classrooms, post-secondary learners must independently self-regulate throughout their coursework by planning how they will complete assignments, setting goals for their learning within the course, metacognitively monitoring their performance, and reflecting on their academic outcomes (Winne and Hadwin, 1998, 2008; Zimmerman, 2013; Winne, 2018). Learners must continually repeat these processes throughout the semester, adapting their SRL behaviors in response to changing internal (e.g., motivation) and external (e.g., increased use of technology-driven instructional tools) factors as they navigate required academic tasks (Winne and Hadwin, 1998, 2008). Students who engage in these behaviors generally exhibit positive academic outcomes; however, many students do not inherently possess effective SRL skills (Winne and Hadwin, 2008; Winne and Azevedo, 2014), which may negatively impact their ability to master the required academic content. For this reason, it is important to investigate students’ SRL behaviors and how they unfold over time, as well as how those behaviors are impacted by shifting factors like course difficulty and students’ own motivation.

There are several data channels that can be used to measure SRL during learning. This includes (but is not limited to): (1) log files of students’ clickstream actions implemented during learning (e.g., mouse clicks to make metacognitive judgments, keyboard entries demonstrating note taking, or student learning analytics of course navigation behaviors; Ochoa and Wise, 2021; Taub et al., 2021), (2) self-reports gauging students’ perceived use of strategies (e.g., MSLQ; Pintrich et al., 1991, SRSI-TRS (Self-Regulation Strategy Inventory-Teacher Rating Scale); Cleary and Kitsantas, 2017), (3) eye tracking to capture visual attention on different elements of a user interface (e.g., inspecting texts and diagrams or other areas of interest; Catrysse et al., 2018; Taub and Azevedo, 2019; Lallé et al., 2021), (4) concurrent think-aloud protocols to record students’ verbalizations (e.g., utterances of a judgment of learning or feeling of confusion; Greene and Azevedo, 2009; Greene et al., 2018; Engelmann et al., 2021), or (5) videos of facial expressions of emotional states to capture the impact of emotions on learning processes (e.g., emotion variability during phases of SRL or impact of emotions on the use of cognitive and metacognitive processes; Li et al., 2021; Taub et al., 2021).

As outlined in Azevedo and Taub (2020), there are advantages and disadvantages for collecting each type of data to examine SRL (see Azevedo and Taub, 2020). Since our paper focuses on trace data, our remaining review will focus on considerations related to using both clickstream data and self-report measures to investigate learners’ SRL behaviors and related contextual factors in online learning settings. There are several strengths for log files; these data are a record of all student actions during learning that are automatically collected *and* timestamped by a system (such as an online learning environment). We can also determine sequences of actions that are time- or event-based. Finally, and arguably most importantly, they are easy to understand and analyze. However, log files require a level of researcher inference making to interpret what behaviors students are engaging in (e.g., are actions indicative of making a plan or metacognitive judgment?) when using these data. Therefore, including screen recordings would provide more contextual information of what elements were on screen during these actions. In contrast, self-reports are a direct measure of student perceptions, thereby not requiring researchers to make inferences of students’ intentions when filling out surveys. However, using self-reports relies on student perceptions as opposed to their behaviors, leaving researchers unaware if students are accurately reporting their actions or beliefs due to possible experimenter bias or a lack of student awareness of behaviors. By utilizing the two data channels (e.g., trace data and self-report measures), researchers interested in investigating SRL behaviors can generate a richer picture of learners’ behaviors within online environments, merging learners’ perceptions and their recorded actions in a complementary manner that stands to mitigate some of these issues.

Recent attention to SRL processes within the learning analytics (LA) community has provided new methods with which to identify real-time SRL behaviors using the aforementioned data channels. Clickstream data (i.e., log files) generated from students’ interactions within blended and online learning environments have gained popularity in this area as a non-intrusive way to capture extensive amounts of granular data, providing the means with which to investigate learners’ behaviors as they unfold across a learning task (Siadaty et al., 2016; Winne, 2017; Saint et al., 2020a). Emerging methods like process mining, sequence mining, and temporal analytics offer new ways to utilize this data channel to capture and analyze students’ SRL behaviors while highlighting the dynamic, contextualized nature of these processes. These methods allow for the interpretation of learners’ real-time behaviors with increased granularity (Azevedo, 2014), tracking changes in self-regulatory behaviors more objectively than traditional self-report measures alone. Despite these benefits and the increased use of log-file data to capture and interpret learners’ SRL behaviors, it is important to note limitations related to the use of this data channel in SRL research. Ongoing challenges in interpreting trace data include inconsistencies in the data produced across different learning management systems, a lack of consensus on what constitutes the optimal levels of data granularity to accurately interpret SRL behaviors, and the absence of a unified SRL theory or framework for this line of research (Winne, 2017). These challenges can be addressed through continued investigations that utilize theoretically grounded interpretations of trace data alongside additional data channels, such as self-report measures or concurrent think-aloud protocols.

As an unprecedented number of higher education students continue to be impacted by the COVID-19 pandemic and related shifts to online learning, researchers now have increased access to large amounts of clickstream data generated within online learning environments and a concurrent need to better understand the factors impacting learners’ success in online course work (Zhang et al., 2021). Continued analysis of learners’ clickstream data, in combination with additional data streams, such as self-report measures, can provide SRL and LA researchers with a deeper understanding of learners’ behaviors when engaging with online content, the contextual factors that may impact those behaviors throughout the course, how those elements work to change students’ SRL processes over time, and the resulting academic outcomes, providing needed guidance for the ongoing development of online and blended learning environments (Marzouk et al., 2016; Winne, 2017).

While existing studies have analyzed the occurrence of micro and macro SRL processes in online learning environments (Siadaty et al., 2016; Saint et al., 2020b; Fan et al., 2021), more research is needed to highlight the dynamic nature of these behaviors, including how learners’ SRL strategies are impacted by temporal changes in internal and external conditions, such as course content difficulty and individual motivation. It can be assumed that successful SRL in online courses requires students to continuously (and often independently) judge and adapt their cognition and metacognition in accordance with shifting internal and external conditions (Winne and Hadwin, 2008), an added component of SRL that makes these processes particularly challenging for post-secondary students who do not possess effective self-regulation skills. By revealing the temporal changes in learners’ SRL behaviors through analysis of clickstream data through lenses provided by theories, such as COPES model of Winne and Hadwin (1998, 2008) and the 2 × 2 achievement goal framework (Elliot and McGregor, 2001), researchers can gain significant insight into how students dynamically adapt their SRL strategies in response to changing contextual conditions, and how their processes unfold across an entire semester.

Building upon existing LA SRL research, this study utilized a combination of hierarchical clustering, process mining, and sequence mining techniques to analyze students’ clickstream data and investigate how learners’ SRL behaviors temporally unfolded throughout a semester-long blended learning physics course. Furthermore, this study interprets these behaviors through the lens provided by COPES model of Winne and Hadwin (1998, 2008), which allows for the examination of relationships between learners’ SRL behaviors and changes in external and internal conditions, highlighting the multifaceted nature of learners’ strategy use within a large post-secondary STEM course. The results of this study provide insight into the measurement and analysis of temporal SRL behaviors, as well as the relationship between those behaviors and additional relevant conditions, such as learners’ achievement motivation profiles and academic outcomes.

### Theoretical Frameworks

Given the many factors (both internal and external) that stand to impact learners’ behaviors in online and blended learning environments over time, it is important to consider SRL behaviors as they relate to additional conditions, such as affective, metacognitive, and motivational processes (Azevedo and Taub, 2020). For this reason, we utilize the COPES model of SRL which considers both the multifaceted and temporal nature of SRL behaviors (Winne and Hadwin, 2008). In addition, we investigated the impact of learners’ reported motivation on SRL behaviors (Cleary and Kitsantas, 2017) through the lens of 2 × 2 achievement goal orientation framework of Elliot and McGregor (2001). In combining these frameworks, we aim to highlight the dynamic, interwoven nature of SRL behaviors and motivation.

#### Winne and Hadwin COPES Model

In this study, COPES model of Winne and Hadwin (1998, 2008) was used to interpret student clickstream data due to the model’s focus on the impact additional factors, such as motivation, have on learners’ SRL behaviors over time. The COPES model (Winne and Hadwin, 2008) describes SRL as a series of events that unfold over time, an important distinction for the temporal analysis of students’ clickstream event traces produced while learning within dynamic contexts like online learning environments. The COPES model posits four phases of SRL in which self-regulating students are actively and repeatedly generating perceptions of an academic task (Phase 1), defining goals and plans related to the completion of that task (Phase 2), enacting planned study tactics (Phase 3), and adapting their plans and future goals based on metacognitive judgments of how well their operations and products aligned with their goals (Phase 4). Within each of these phases, the researchers further describe features related to how a student COPES with a task, an acronym that illustrates how the *Conditions* (e.g., internal and external contexts for students’ work), *Operations* (e.g., cognitive processes enacted by students), *Products* (results of the enacted operations), *Evaluations* (e.g., information based on the created products), and *Standards* (e.g., criteria used to monitor products) of a given task further influence students’ learning and enacted SRL behaviors. Within the context of self-paced blended and online learning environments, this means that a student’s SRL behaviors are continually impacted by a range of internal and external factors, including task conditions like low prior content knowledge or evaluations of products like quiz scores (Winne and Hadwin, 2008). As learners in self-paced blended and online learning environments independently navigate these recursive phases and related judgments of their learning over time, it is important to consider how the additional impact of changing internal and external factors, such as motivation, work to shape students’ SRL behaviors.

#### Achievement Goal Orientation

The COPES model further emphasizes the impact of internal and external factors, such as content difficulty and motivation, on students’ behaviors during each of the four phases of SRL (Winne and Hadwin, 2008). Through this lens, contextual factors like achievement motivation can provide added insight when investigating learners’ SRL behaviors. The 2 × 2 achievement goal orientation framework has been widely used to examine learners’ motivation across academic contexts and provides a complementary theoretical perspective with which to further consider the relationship between learners’ motivation and their enacted SRL behaviors (Kaplan and Maehr, 2007; Elliot et al., 2011; Cleary and Kitsantas, 2017). The framework defines four distinct achievement goal orientations that differ in definition (mastery and performance) and valence (approach and avoidance), each with a unique set of antecedents and outcomes (Elliot and McGregor, 2001). In this framework, learners who are mastery oriented are motivated by content mastery while performance-oriented learners are driven by peers’ perceptions of their academic competence, with the added valence component of approach (positive) and avoidance (negative) further delineating differences in goal orientation (e.g., a student with a performance approach orientation is believed to be motivated by a desire to appear competent while someone with a performance avoidance orientation wants to avoid appearing incompetent). The resulting goal profiles [i.e., mastery approach (MAP), mastery avoidance (MAV), performance approach (PAP), and performance avoidance (PAV)] have been widely researched in a variety of academic settings, with approach-based goal orientations frequently linked to desired academic outcomes (Linnenbrink and Pintrich, 2002; Van Yperen et al., 2014) and focus on success (Elliot et al., 2011). The Achievement Goal Questionnaire-Revised (AGQ-R) is still frequently used to determine learners’ self-reported goal orientations (Elliot and Murayama, 2008). The AGQ framework and its associated goal orientations have been used to examine relationships between learners’ achievement motivation, SRL behaviors, and academic outcomes, but only recently have researchers begun to explore the temporal dynamics of these relationships.

In combining these two theoretical perspectives, we aim to examine how students’ SRL behaviors unfolded throughout the online learning course as well as how those behaviors were impacted by external factors, such as learner motivation.

### Literature Review

There is a lot of research using multichannel multimodal data to examine SRL (Azevedo et al., 2018; Azevedo and Taub, 2020), despite some potential limitations (discussed above), that affords us the opportunity to investigate SRL processes and behaviors in a more dynamic way. Specifically, trace data or learning analytics can be used to capture student behavior throughout a semester during online learning (Winne, 2017). We focus the literature review of this paper on data and analyses investigating the changing nature of SRL as the goal of the current study was to contribute to this field of emerging research by using some established LAs methods, such as process mining. In addition, our paper also contributes to the field of SRL by examining the temporal nature of factors that impact SRL (Cleary and Kitsantas, 2017), as motivation (AGQ) is not typically examined more than once during a learning session.

#### Temporality of SRL and AGQ

Historically, SRL research has relied on self-report measures to identify and examine learners’ use of SRL processes within academic contexts, viewing SRL as a trait rather than an event that unfolds during learning (Azevedo, 2014; Winne, 2017). These measures are inherently subjective (respondents may not be conscious of the SRL strategies they use) and are often administered at a single time point within a study (e.g., after a student completes an academic activity), which may fail to capture the dynamic nature of learners’ SRL processes. Recent shifts toward the use of multimodal data in SRL research have allowed for more detailed investigations of SRL, with current works using advanced data channels and analyses like multilevel modeling to highlight the dynamic, interrelated nature of learners’ SRL behaviors (Taub et al., 2017; Winne, 2019; Li et al., 2020). The influence of these advances can be seen within current LA research using large-scale data sets to analyze SRL behaviors, with recent work considering the temporal dynamics of those students’ processes (Saint et al., 2020b). However, continued investigation is needed to establish best practices for using temporal analytics to analyze SRL behaviors (Molenaar and Järvelä, 2014; Chen et al., 2018a).

While SRL research continues to benefit from the inclusion of more fine-grained data channels, investigation of learners’ achievement motivation is still largely reliant on data generated from single administration self-report measures (Urdan and Kaplan, 2020). Despite this, the dynamic nature of motivation has prompted researchers to consider the stability of learners’ achievement goal orientation, examining if and how learners’ achievement motivation changes over time (Senko and Harackiewicz, 2005; Fryer and Elliot, 2007; Muis and Edwards, 2009). Fryer and Elliot (2007) argue the adaptive nature of self-regulation, as well as changing internal and external antecedents (e.g., classroom environment and content difficulty), are equally as likely to result in goal stability or change in learners’ achievement goal endorsements, despite the literature’s focus on achievement goals as a fixed personal state. Through this lens, recent studies investigating changes in learners’ achievement goals have used repeated self-report administrations to investigate longitudinal trends in learners’ goal endorsement (Lee et al., 2017; Tuominen et al., 2020). The temporal nature of these studies stands to complement existing analyses of unfolding SRL behaviors, allowing for the incorporation of contextual factors like learner motivation.

#### Analyzing Students’ SRL Behavior Using Process and Sequence Mining

Multiple recent studies have investigated students’ use of SRL strategies by analyzing clickstream data using techniques, such as sequence mining, process mining, and hierarchical clustering. For example, process mining has been used across multiple studies (Siadaty et al., 2016; Maldonado-Mahauad et al., 2018; Matcha et al., 2019; Fan et al., 2021), to identify learners’ interaction strategies, learning tactics, indicators of engagement in SRL processes, and to develop SRL process maps from micro-level SRL processes as a means of comparing learners’ behaviors in response to varying interventions and course structures. Sonnenberg and Bannert (2019) also utilized conformational checking (a process mining technique) to identify the stability of metacognitive prompts of SRL behavior. Most recently, Saint et al. (2020b) proposed the Trace-SRL framework for analyzing clickstream data by multiple levels of trace clustering and process mining.

Within many of the existing studies, the clickstream data being analyzed were collected from online learning environments that provide a rich variety of event traces, ranging from the number of problem attempts to how frequently learners access dashboards. Students also had relatively high levels of freedom to access different course components in their preferred order. Under those conditions, students’ different SRL strategies are likely to produce event traces with distinct event types and event orders, which makes it easier for both the interpretation and the clustering of event traces.

However, clickstream data from other popular online learning systems are often markedly more restrictive, containing fewer event types and less variability in event orders. For example, homework platforms or intelligent tutoring systems may require students to complete assignments in a pre-determined order that is pedagogically beneficial. In addition, certain events, such as checking the dashboard, may not be recorded in the data set or are stored in a different data set that may not be readily available to the researcher.

In those cases, a different analysis scheme is needed to extract information about students’ SRL behaviors from clickstream data that contain a much smaller set of event types and possible event orders.

### Current Study

The goal of the current study was to investigate students’ SRL behaviors and self-reported achievement goals, as well as how each of them changes throughout the semester in a college-level physics course that has students complete online learning modules (OLM). We argue that based on the COPES model of SRL (Winne and Hadwin, 1998, 2008), SRL is a cyclical process that consists of a series of events that temporally unfold during learning and studying. Therefore, students will demonstrate different self-regulatory events, such as motivational processes, during learning with the online learning modules over a period of time under changing external conditions.

#### Research Questions

To address if and how students’ SRL and motivational processes changed throughout the semester, we posed the following research questions for our study:

1. What are the different types of SRL processes students employ in a self-paced online learning environment?
2. To what extent do students’ SRL behaviors and AGQ responses change over the semester?
3. How do changes in observed SRL behavior and AGQ responses relate to students’ learning outcome?

#### Mastery-Based Online Learning Modules

The current study examines students’ SRL behavior in a mastery-based OLM system, designed based on principles of mastery-learning (Bloom, 1968; Kulik et al., 1974; Gutmann et al., 2018) and deliberate practice (Ericsson et al., 1993, 2009).

**Figure 1:**
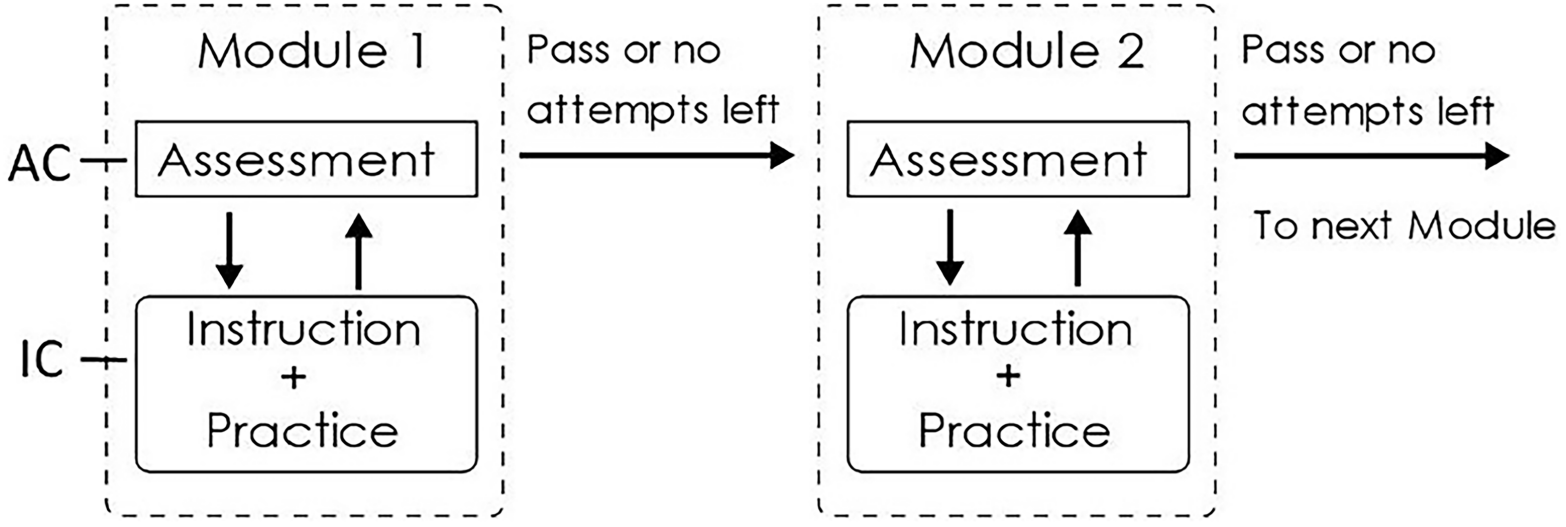
Overview of online learning modules (OLMs).

An OLM is a standalone online learning unit that combines assessment, instruction, and practice, centered around one or two basic concepts, or developing the skills to solve one kind of problem. Each OLM (see Figure 1) is designed to be completed by the average student in about 5–30 min, depending on their incoming knowledge. Each OLM consists of an assessment component (AC), which tests students’ content mastery in 1–2 questions, and an instructional component (IC) with instructional text and practice problems on the topic. Upon accessing a module, students are shown the learning objectives of the current module and are required to make an initial attempt on the AC before being allowed to access the IC. If the first attempt fails, students can make additional attempts either immediately after the first or after interacting with the IC. This design is motivated by both the “mastery-learning” format that allows students who are already familiar with the content to proceed quickly to the next assignment, and by the concept of “preparation for future learning” intending to improve students’ learning from the IC by exposing them to the questions first. It also provides better interpretability of student log data (Chen et al., 2018b) and allows for measurement knowledge transfer between consecutive modules (Whitcomb et al., 2018, 2021; Chen et al., 2019).

A number of OLM modules form an OLM sequence on a more general topic typically covered over a period of 1 or 2 weeks in the course. Students are required to pass the AC or use up all attempts on one OLM before moving onto the next in the same sequence. A typical OLM sequence consists of 5–12 modules that are assigned as self-study homework for students to complete over a period of 1–2 weeks.

#### Multilevel Hierarchical Clustering

In order to observe the changes in students’ SRL strategy from log data collected from the OLM platform, we developed a novel analysis scheme involving three consecutive clustering operations on three consecutive levels of data granularity:

##### Level I

Clustering of individual events: Prior research on OLMs (Chen et al., 2020; Garrido et al., 2020) has shown that an abnormally short assessment attempt is likely the result of random guessing or answer copying, perhaps indicative of the student adopting a performance avoidance goal. Therefore, the main goal of event-level clustering was to distinguish between abnormally short guessing attempts and normal problem-solving attempts. This was achieved by fitting the log distribution of attempt duration on each AC with finite mixture modeling (FMM), which can be seen as clustering based on a single continuous variable (event duration), following a similar procedure outlined in (Chen et al., 2020). The same method was also applied to identify and exclude very short study events which likely originated from a student clicking through the instructional contents without meaningfully interacting with them.

##### Level II

Clustering event traces on a single module: To identify the main strategies that students adopt when interacting with individual OLM modules, we partitioned students’ event traces on a single module into multiple “module-level clusters” by hierarchical agglomerative clustering. Different from most existing trace clustering methods that use the “edit distance” as a metric for calculating the dissimilarity between event traces, the current analysis calculates the dissimilarity based on a set of derived features. Those features were selected according to a model of student interaction with OLMs based on the COPES framework of SRL. For each resulting module-level cluster, a causal net was generated using heuristic process mining for 80% of most frequent traces for visual interpretation of the main strategy.

##### Level III

Clustering of module-level cluster traces for entire OLM sequences: As a result of module-level clustering, students’ interaction with an entire OLM sequence can be captured as a trace of multiple module-level cluster memberships. We can then partition those traces into sequence-level clusters by conducting hierarchical clustering based on the optimal matching distance between each pair of traces. Using the optimal matching distance preserves the information on the temporal order of the module-level cluster memberships, which enabled us to investigate when students change their interaction strategy in response to change in content difficulty or other factors.

## Materials and Methods

Data used in this study were collected from a calculus-based university introductory physics course taught in the Fall 2020 semester. A total of 251 students (27% female) were initially enrolled in the class. The course was taught asynchronously using pre-recorded lecture videos as the main method for content delivery during the COVID pandemic. Students and instructors interacted *via* messages, posts, and video conferences (for more information of the course design^1^). Students were required to take a total of seven 20-min quizzes during the semester.

A total of 70 OLMs consisting of nine sequences were assigned as online homework and self-study material. Each OLM sequence was assigned for students to complete over 1–2 weeks. Students could earn extra credits by completing some OLMs earlier than the due date, as explained in more detail in Felker and Chen (2020).

### Data Collection

We collected data from the following channels: (1) self-reported achievement goals *via* the Achievement Goals Questionnaire-Revised, and (2) event data as students interacted with the OLM. Students were given the AGQ survey as an optional activity in the course with no extra credit nor any other incentives associated with completing them. Students enrolled in the course were presented with an informed consent at the beginning of the course, which explained that their interaction with the course, including surveys, will be used for research purposes, and their identity would not be revealed in the research.

#### Achievement Goal Questionnaire-Revised

The AGQ-R was administered at three different points throughout the course. The 12-item questionnaire measures students’ achievement goals through four subscales, with each subscale representing one of four achievement goal orientations (see section “Achievement Goal Orientation”; Elliot and McGregor, 2001). Students were asked to rate their agreement (from 1 = “*strongly disagree*” to 5 = “*strongly agree*”) to each of the statements as a means of measuring their goals and expectations as they related to the course. Confirmatory factor analyses support the continued use of the AGQ-R to measure achievement goal orientation within academic contexts [*χ*^2^(1.63) = 78.32, *p* < 0.01, CFI = 0.99, IFI = 0.99, RMSEA = 0.053; Elliot and Murayama, 2008]. Additionally, all four subscales were found to have high levels of internal consistency [mastery approach (=0.84), mastery avoidance (=0.88), performance approach (=0.92), performance avoidance (=0.94); Elliot and Murayama, 2008].

Each AGQ-R administration coincided with one of the three course sequences, resulting in three sets of questionnaire responses that represented students’ achievement goal orientations at roughly the beginning, middle, and end of the term. Student response rates declined slightly from the beginning of the term (*n* = 248) to midterm (*n* = 238) and fell dramatically by the end of the course (*n* = 40). For this reason, only scores from the first and second survey administration were included in the analyses.

#### Online Environment and Event Data

The OLM modules were created and hosted on Obojobo Learning Objects Platform, an open-source online learning platform developed by the Center for Distributed Learning at the University of Central Florida. In the current iteration, the assessment component of each OLM contains 1–2 multiple choice problems and permits a maximum of five attempts. The first three attempts are sets of isomorphic problems assessing the same content knowledge with different surface features or numbers. On the fourth and fifth attempts, students are presented with the same problems in the first and second attempts, respectively, and are awarded 90% of credit. The instructional component of each module contains a variety of learning resources including text, figures, videos, and practice problems. Each OLM sequence contains between 3 and 12 OLMs, which students must complete in the order given, with completion defined as either passing the assessment or using up all five attempts. Each OLM sequence is assigned over a period of 1 or 2 weeks depending on the length of the sequence. Readers can access example OLMs at https://canvas.instructure.com/courses/1726856.

For the current study, we extracted student event data from clickstream log files from three OLM sequences: Sequence 1: Motion in 1 Dimension, Sequence 6: Mechanical Energy, and Sequence 9: Angular Momentum. The three sequences were assigned to students during week 2, week 7 and 8, and week 14 of the semester, respectively. They consist of a total of 26 modules, and the resulting data set contains a total of 5,960 traces. In addition, all records after the first passing attempt or after the last attempt were truncated for simplicity of analysis, since there were significantly fewer records after passing or using up all attempts, and most of those events took place before an exam (Chen et al., 2020).

### Data Coding and Scoring

#### AGQ Change Scores

Changes in students’ aggregate scores for each subscale across the first and second administration of the AGQ-R were calculated using the reliable change index (RCI; Jacobson and Truax, 1991; Fryer and Elliot, 2007). The RCI provides a standardized method for categorizing participants by the amount of change in their scores across two test administrations given at separate time points. The RCI formula below was used to compute an RCI score for each of the four achievement goal profiles, allowing us to examine the level of change in students’ AGQ-R scores between the beginning of the semester (*x*_1_) and the middle of the semester (*x*_2_), resulting in four RCI scores that correspond with the four established achievement goal constructs for each participant (Jacobson and Truax, 1991; Elliot and McGregor, 2001).


$$RC=\frac{x_{2}-x_{1}}{S_{diff}}$$

The standard error of difference between students’ AGQ-R responses at the beginning and middle of the term (*S*_diff_) was calculated using the method discussed by Jacobson and Truax (1991).


$$S_{diff}=\sqrt{2{\left(S_{E}\right)}^{2}}$$

Resulting RCI scores allowed for the categorization of students’ change in goal endorsement over time for each of the four goal orientation profiles [MAP (*M* = −0.4, SD = 0.99), MAV (*M* = 0.211, SD = 0.91), PAP (*M* = −0.03, SD = 1.01), PAV (*M* = −0.15, SD = 0.96)].

#### Log File Event Processing

Students’ clickstream log data collected from the Obojobo learning platform was first processed into attempt events and study events. An attempt event starts when the student enters the assessment page of the module and ends when the student clicks the submit button on the assessment page. During this period, the student is unable to navigate to any other pages in the current module or to other modules. The duration of the attempt event is defined as the time between those two clicks minus the duration of: (1) when the browser window is either closed or minimized, or when another window is in focus and (2) any non-active duration beyond 10 min. A “Pass” event is added after an attempt event only if the student correctly answers all questions in the assessment on that given attempt. A study event starts when the student clicks on any page in the instructional component of the module and ends when the student clicks on the last record before a new attempt event is initiated. In other words, a study event includes all the interaction with the instructional component between two attempt events. The duration of the study event is calculated as the sum of all the time spent interacting with each instructional page, minus the duration of inactive periods (explained above). In the current analysis, a small fraction of events that took place after the “Pass” event were excluded from the analysis.

#### Event-Level Trace Clustering

At this stage, abnormally short attempts on the assessment component (AC) of a given OLM were distinguished from normal AC attempts by fitting the log duration distribution of all attempts on a single module using FMM. FMM is a model-based clustering algorithm that divides a population into subgroups according to one or more observable characteristics by fitting the distribution of characteristics with a finite mixture of normal or skewed probability distributions. When two or more distinct problem-solving behaviors are present, the log attempt duration distribution can be fitted with the sum of two or more distributions, with the shortest distribution corresponding to abnormally short attempts. In the current study, we fit the log duration of each assessment attempt using either normal or skewed distribution models using the R package mixsmsn (Prates and Cabral, 2009), following the fitting procedure described in detail in the appendix of a previous study (Chen et al., 2020). In the case when a single component distributed was the best fit for the duration, the cutoff was set as either 2 standard deviations below the mean duration, or 15 s, whichever was longer (Guthrie et al., 2020).

The main reason for using a different cutoff for different problems, rather than using a single, uniform cutoff is because certain conceptual problems require significantly less time to solve than numerical calculation problems. In one previous study (Chen et al., 2020) it was found that the mean duration for answering certain conceptual problems can be as short as 30 s. Using an individualized cutoff avoids accidentally categorizing half of the class as making a “short” attempt on those conceptual problems. On the other hand, certain numerical problems also have longer and more sophisticated problem text, and students who are making a decision to guess or answer copy after reading the text might also take longer. On those problems, short attempts may also include students who solved the problem using incorrect methods that are significantly faster than the correct method.

We also conducted mixture-model fitting of the combined log duration of all study events from all modules in the data set to determine the cutoff time between normal study events and very short study events that were likely the result of students clicking through the instructional pages. Unlike short assessment attempts, which could include cases in which the students read the problem body, the very short study events identified using this method predominantly consist of students who clicked through the pages without meaningfully interacting with the materials. Since those study events are content-independent, the fitting is conducted on all study events, which could amplify the frequency of content-independent actions. Note that the current analysis methods do not distinguish between short interaction and extensive interaction with learning materials. This is because short interactions could result from students actively searching for information that they need, which reflects high levels of self-regulation.

#### Module-Level Clustering

As a result of the event-level clustering, each student’s interaction with a given OLM was represented by a trace of either normal or short attempt events and study events that are longer than the minimum duration. Study events shorter than the minimum duration are excluded since the majority of those are “click-through” events with no meaningful interaction with the material.

Each attempt is treated as a separate event and labeled as “Attempt_N” with N being the attempt number. Short attempts are labeled as “Attempt_N_S” to distinguish from normal attempts. For example, a trace of {Attempt_1_S, Study, Attempt_2, Attempt_3} indicates that the student took three attempts on the OLM, with the first attempt being a short attempt, and took a study session (longer than the minimum cutoff) between attempts 1 and 2. Hierarchical agglomerative clustering using Ward’s method was performed *via* the R package cluster (Maechler et al., 2021) on traces from all three selected OLM sequences, with each trace treated as a data entry. The distance metric that determines the distance between any two traces, which is central to the clustering algorithm, was determined by a set of features for each trace selected based on the COPES SRL framework of Winne and Hadwin (1998, 2008), explained below.

**Figure 2:**
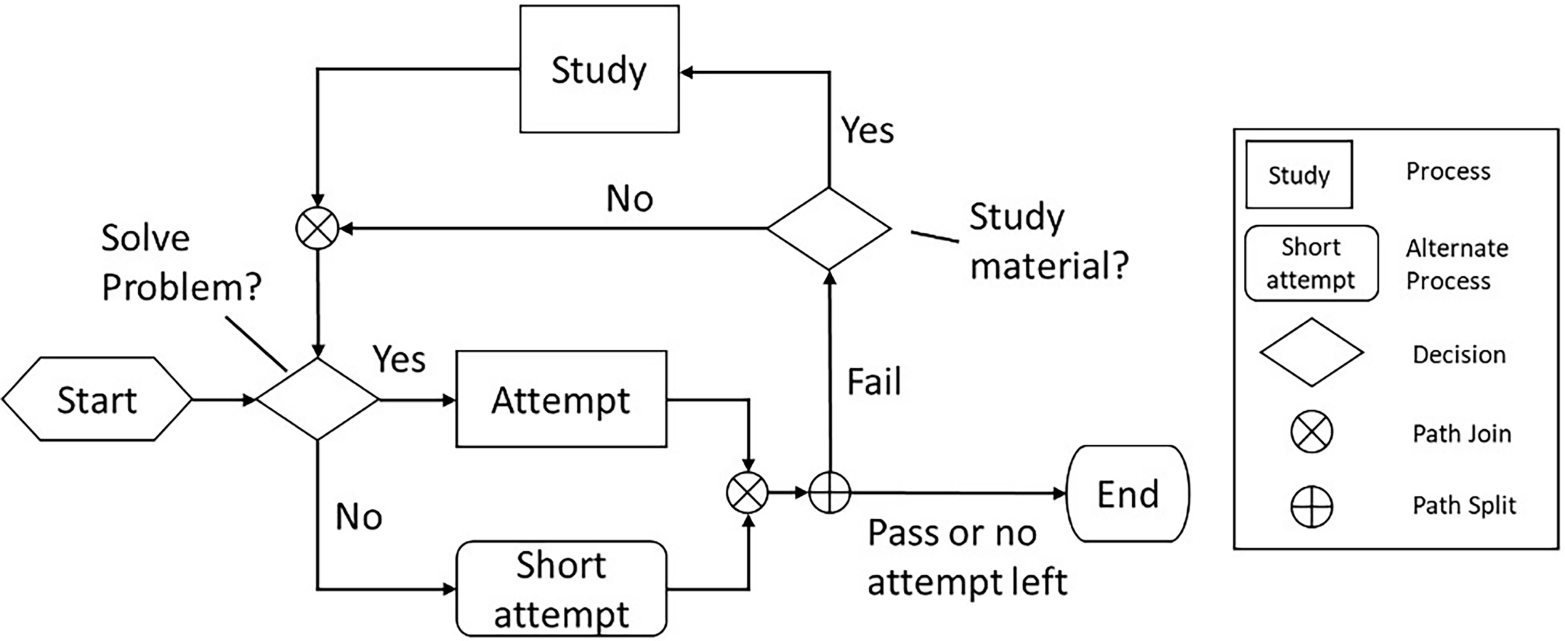
Interaction process within a single OLM.

A student’s interaction process with a single OLM can be summarized in the flowchart shown in Figure 2. For each OLM, students start with the mandatory first attempt on the assessment. If the attempt fails, then the student can either study the instructional material or immediately make another attempt, until they either successfully pass the assessment or use up all five attempts. During the process, students are presented with two tasks: a required task, which is to answer the problem in the assessment component, and an optional task, which is to study the learning material. Students needed to make two types of decisions: (1) whether to seriously engage in problem-solving on a given attempt (resulting in a normal length attempt) or to make a guess (usually resulting in a short attempt) and (2) whether to engage with the study material if the previous attempt fails.

Using the four recursive phases of SRL presented in COPES model of Winne and Hadwin (1998, 2008) we propose six features that capture students’ interactions with the OLMs and their associated SRL processes:

1. Total Number of Assessment Attempts (nA): The total number of assessment attempts reflects the quality of a student’s enacted plan of action on both problem-solving tasks and studying tasks. In general, passing on fewer attempts indicated students entered with high subject matter knowledge, engaged in successful self-instruction, or both.
2. Number of Attempts Before Study (nY): When or whether to access study materials can be influenced by learners’ planning or adaptation. Students who access study materials are likely generating reflective self-evaluations and continually engaging in planning based on their judgments of conditions like existing content knowledge or previous assessment scores. Continued assessment attempts and subsequent access of study materials may indicate a student has reflected on prior performance and is engaging in setting new goals and planning based upon a reassessment of their strategy use following failed assessment attempts. In the current data, 90% of study events took place after a first attempt.
3. Fraction of Short Attempts Among All Attempts (fS): Since most short attempts likely originate from either guessing or answer copying behaviors, a higher fraction of short attempts signifies limited planning and enacting lower quality study strategies. Many short attempts may indicate low prior knowledge, low self-efficacy, low effort, or limited execution strategies like time management.
4. Is the First Attempt Short (1S): The first attempt is of particular significance as it reflects a strategic choice based upon students’ perception of the task, with the outcome of students’ task analysis determining the amount of time and effort they will dedicate to the mandatory first attempt before accessing associated learning materials. A short first attempt may signal that a student plans to limit the time and energy they devote to the task by guessing. This strategy enactment could also indicate that a student is adapting their strategy use based on previous modules and their perceived self-efficacy within the course. A student who experienced prior frustrations may experience low content self-efficacy, resulting in limited energy or motivation to engage with the course and a short first attempt for subsequent modules. On the other hand, making a short first attempt may indicate the student is aware of their low prior knowledge and wants to access the material as quickly as possible because they know they need to learn the content before answering any quiz questions.
5. Is the Last Attempt Short (lS): The last attempt is also of particular significance since it is the passing attempt in all event traces, except for those with five failed attempts. A short final passing attempt may signify limited monitoring during the enactment, with students struggling to effectively activate relevant task strategies during content learning and problem-solving tasks. This feature may also indicate adaptation based on learners’ negative interactions with prior assessments modules in the course, resulting in limited motivation to devote significant time to the final assessment attempt.
6. Did the Student Abort the Module (Ab): This feature represents a small number (22 out of 5,960) of event traces that ended on a failed attempt prior to assessment attempt 5. Those traces exist either because the student aborted the module, or because of corrupted data logs. This behavior may indicate that a student’s deliberation produced a negative self-evaluation in which they saw no means of successfully completing the module, leading them to adapt by discarding the learning task prior to a successful assessment attempt.

Since features 1, 2, and 3 are numeric while features 4, 5, and 6 are binary, the distance metric between two event traces is computed using the Gower dissimilarity coefficient. The Gower dissimilarity coefficient allows for the assignments of different weights to different features. We tested four different sets of feature weights. The first three sets emphasize the task perception, goal setting and planning, enactment, and adaptation phases respectively, while the last set puts equal weight on all features.

The best cluster structure, as judged by the maximum average silhouette value described below, was produced by the set of feature weights emphasizing the task perception and goal setting and planning phases, where the weights for nA and lS are set to 0.5 and all other weights set to 1.0.

##### Selecting the Optimal Number of Clusters

Since agglomerative clustering produces a tree structure of all possible numbers of clusters, we choose to determine the optimum number of module-level clusters based on the average silhouette value of each cluster. In short, the average silhouette value is a measure of the ratio of intra- and inter-cluster variability which is described by Rousseeuw (1987). A larger silhouette value indicates tighter cluster structure. Theoretically, the optimal number of clusters is chosen to maximize the average silhouette, as it indicates that the variability within clusters is minimized compared to the variability between clusters, thus being well defined.

However, in practice, the current data set of 5,960 traces contains only 53 unique traces. As a result, the average silhouette will always reach the global maximum at or near 53 clusters, as the within cluster variability approaches zero. Therefore, we instead chose the number of clusters according to the local average silhouette maximum under 10 clusters, since more than 10 clusters caused significant difficulties in the interpretation of observed clusters as the differences became trivial. Of all the four feature weights tested, the set that emphasized the forethought phase resulted in a local silhouette value maximum below 10 clusters.

To visualize the main characteristics of each identified module-level cluster, we generated causal nets on the most frequent 80% of traces, by applying the heuristic mining algorithm using the R package heuristicsmineR (Weijters and Ribeiro, 2011).

#### Sequence-Level Clustering

Since a student’s event trace interacting with a single OLM is classified into one of several module-level clusters (see above), their interaction with an entire OLM sequence of *n* modules was captured by a sequence-level trace of *n* elements in the form of {*m*1, *m*2…*mn*}. Each element *m*_1_ is represented by a number indicating the module-level cluster that the student’s event trace on module *i* belongs to. We performed hierarchical agglomerative trace clustering on the sequence-level traces for each of the three OLM sequences separately. The dissimilarity between two traces was calculated using the optimal matching distance *via* the TRATE method, as it takes into account the local ordering of states. Since each student contributes one trace per sequence to the data set, the sequence-level clusters reflect the strategy adopted by each individual student on a given module sequence.

The number of s-clusters for each sequence was determined by maximizing the average silhouette value between 2 and 10 clusters. In the case that the maximum average silhouette is two clusters, but a second maximum exists for a higher number of clusters, then the higher number of clusters is selected to display relatively rare but distinct strategies.

## Results

### Research Question 1: What Are the Different Types of SRL Processes Students Employ in a Self-Paced Online Learning Environment?

For this research question, we first outline the results of event-level finite mixture modeling to distinguish between short and normal assessment attempts, followed by a description of student behavior clusters at the module-level and the sequence-level. Module-level clusters are behaviors students engaged in while completing an individual module within the course. Sequence-level clusters outline behaviors across multiple modules in the same OLM sequence.

#### Event-Level FMM Fitting

Of the 26 modules included in this study, the log attempt duration distribution on the assessment component (AC) of eight of the modules was fitted with one component FMM, and the rest are all fitted with 2 or more component FMMs. For four modules, FMM determined the short vs. normal attempt cutoff to be less than 15 s and was adjusted to 15 s. The short vs. normal cutoffs of 16 modules were between 15 and 60 s, four modules were between 60 and 120 s, and two modules had cutoffs beyond 120 s. Of those two modules, visual examination of the distribution profile found one of the modules to be an artifact of overfitting, and the cutoff was adjusted to 35 s based on best estimates from a previous study on OLMs (Chen et al., 2020). In general, ACs of modules involving numerical calculation problems had longer cutoffs compared to those involving conceptual questions, indicating that the short attempts identified likely include “educated guesses” in which students make a guess after reading the problem, or students solve problems using fast, incorrect methods.

Applying the same FMM fitting method to the distribution of all study events determined the cutoff for abnormally short study event to be at 35 s. Therefore, all study events less than 35 s were deemed to be not authentically interacting with the learning materials and removed from the data set.

#### Causal Nets for Module-Level Clusters

**Figure 3:**
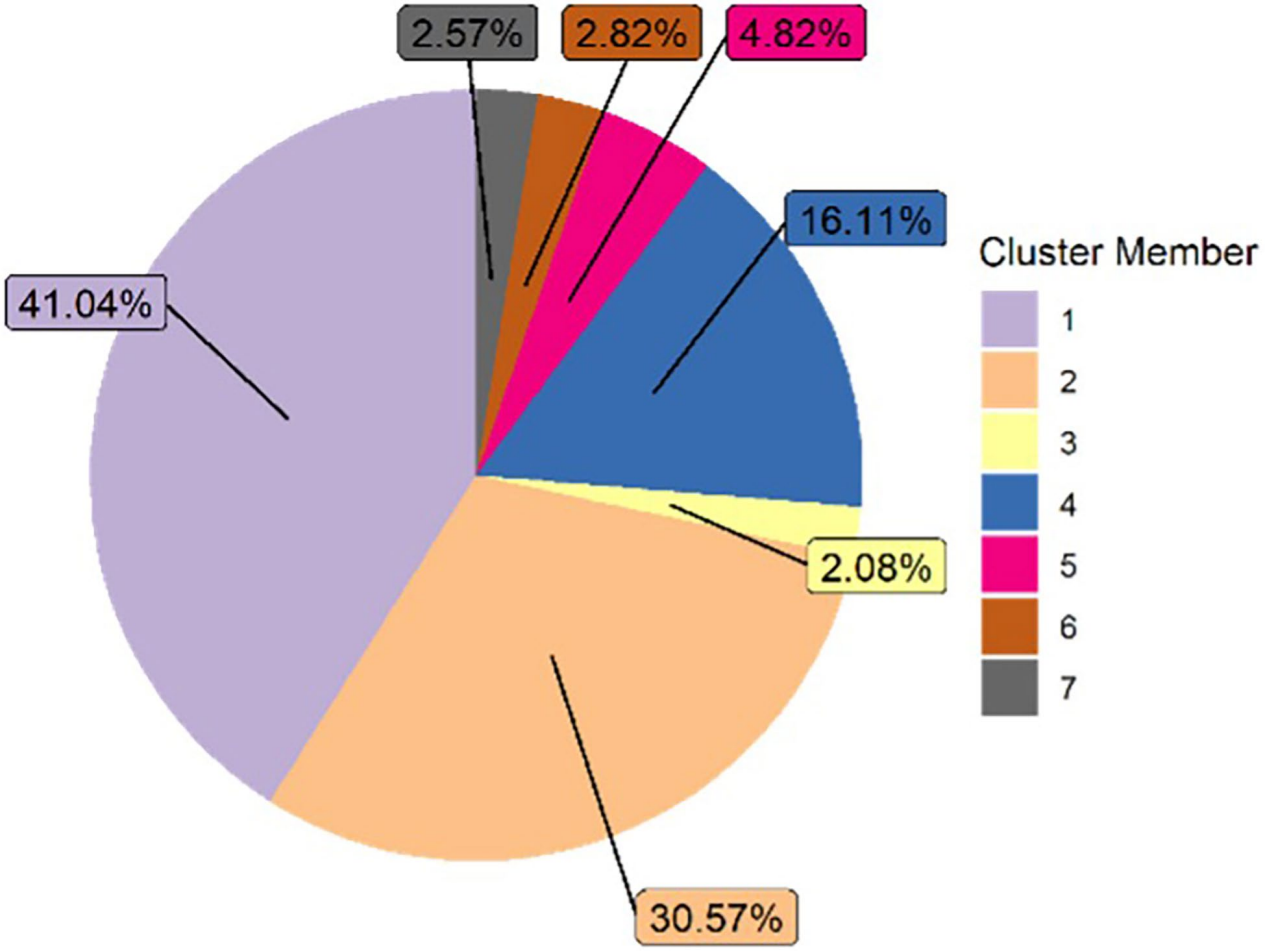
Distribution of causal nets.

We applied heuristic miner, a process mining algorithm (Maechler et al., 2021) on 80% of the most frequent traces of each module-level cluster to capture the main patterns in student behavior through causal nets. Seven types of causal nets were generated, with the frequency distribution plotted in Figure 3 (i.e., the percentage of each causal net represented in the data). As seen in the figure, the most dominant module-level cluster was cluster 1 (*normal first or second pass*), followed by clusters 2 (*attempt, study, attempt, pass*) and 4 (*short attempt and pass*). As such, although we did identify seven different causal nets, in the majority of cases, students’ interaction data can be classified into module-level clusters (or causal nets) 1, 2, and 4.

##### Causal Net 1: Normal Attempts and Pass

**Figure 4:**
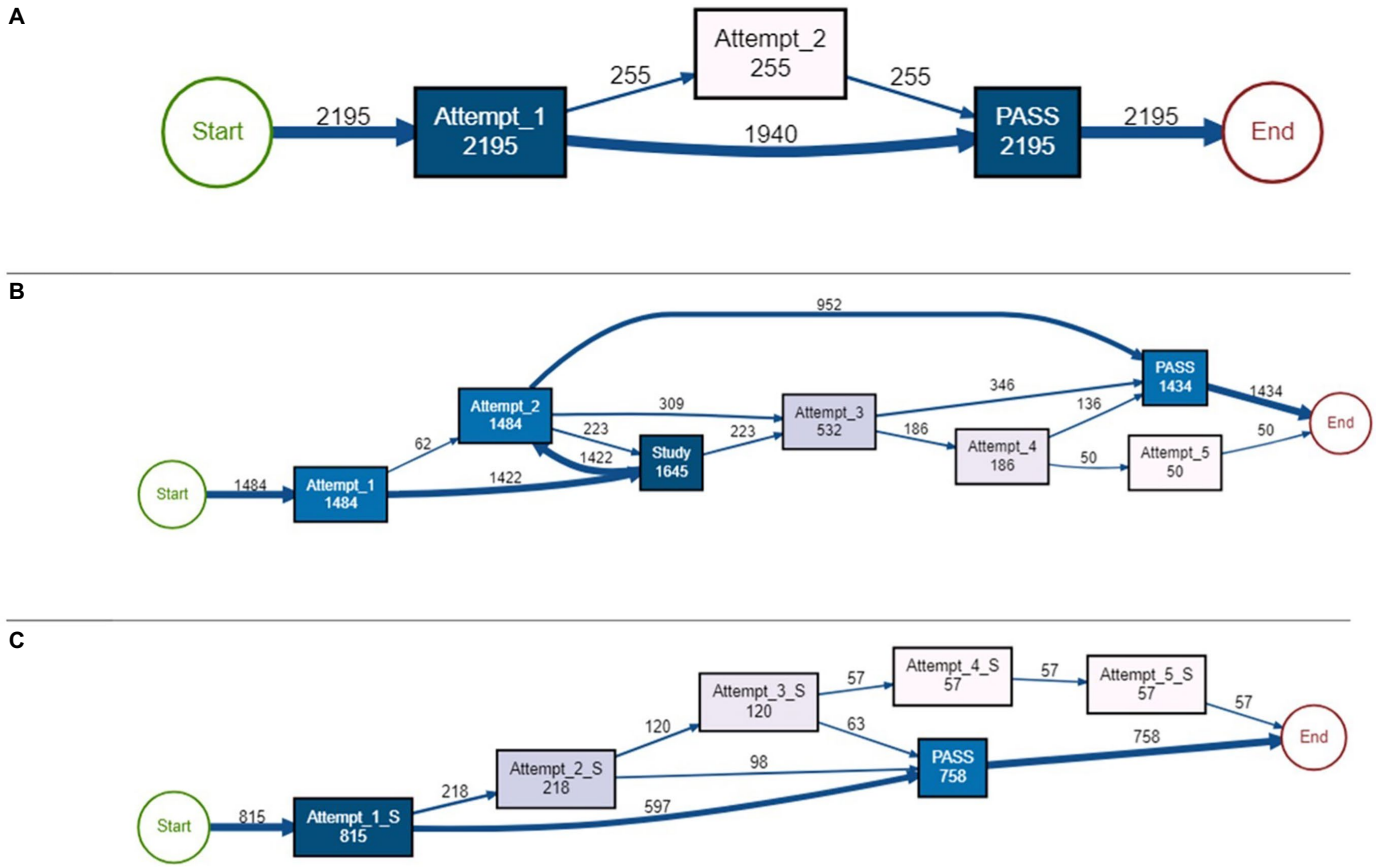
Causal nets. **(A)** Normal first or second pass, Causal net 1; **(B)** attempt, study, attempt, pass, Causal net 2; **(C)** short attempt, pass, and Causal net 4.

In this causal net, students demonstrated beginning the module and passing the quiz on either their first attempt without needing to access the instructional content or on their second attempt. Both types of attempts were normal, indicating students spent an adequate amount of time making these attempts. From a self-regulatory perspective, this can indicate these students were spending time activating their prior knowledge and self-assessing what they already knew about the topic. If they were able to do so effectively and had sufficient prior knowledge of the topic, this could have led to a correct response to the question, as demonstrated by the first successful attempt. If a student needed to make the second attempt, perhaps they did not pay close attention to or misunderstood the question. After spending more time reading through the question and activating more prior knowledge, they ultimately passed. The causal net (see Figure 4A) indicates there were at least 1,940 traces of passing on the first attempt and at least 255 traces of passing on the second attempt, with a total of at least 2,195 traces in this cluster.

##### Causal Net 2: Attempt, Study, Attempt, Pass

In this causal net, most students made a normal failed attempt, followed by studying the course content (at least 1,422 traces), and then either passed the assessment on the second (at least 952 traces), third (at least 346 traces), or fourth (at least 136 traces) attempt. Some students were not able to pass by the fifth attempt. Students who passed on the third or fourth attempt did not return to studying after the failed second attempt; they simply took the quiz again and passed. Regardless of the attempt number, all attempts had “normal” attempt times, meaning students were taking the time to complete the assessment problems, perhaps paying special attention to reading and answering the questions. From an SRL perspective, contrary to the first causal net, these students may have attempted to activate their prior knowledge (as seen in the normal first attempt); however, they did not possess sufficient prior knowledge to pass the assessment, leading them to study the course material. For some students, this study event led to a successful attempt, while others were still unable to pass. Perhaps after reading, students engaged in a judgment of learning (asking if they felt they understood the content) and deemed they now understood the material. Some students made accurate judgments, however other students did not and continued to attempt the assessment without studying the material again. Students who continued to take the quiz might have still felt like they covered enough material, but felt they needed to focus more attention on the question (i.e., continued to make normal, as opposed to short attempts). After three or four attempts, some students did pass the quiz (at least 346 traces after attempt 3 and at least 136 traces after attempt four). There were at least 50 traces of making a fifth attempt, but not passing after this attempt. Therefore, these students were perhaps demonstrating some regulatory behaviors, including planning by activating prior knowledge, making adaptations by accessing the content, then monitoring their performance, even though they did not always pass the assessment. Overall, there were at least 1,434 traces of passing behaviors and 50 traces of non-passing behaviors. See Figure 4B for the causal net of this cluster.

##### Causal Net 4: Short Attempt and Pass

This causal net can be described by the majority of students (at least 597 traces) passing after a short first attempt. Some students continued to make more attempts, but none of the attempts were normal (i.e., all attempts were short attempts). Traces in this cluster did not include any study attempts, nor did it include any normal attempts. From an SRL perspective, these traces could indicate students started the module with low self-efficacy and therefore planned from the onset to pass the assessment (by possibly guessing) as soon as they could. These traces do not demonstrate students were monitoring or making any adaptations to their plans because failed short attempts were always followed by another short attempt, therefore not demonstrating any change in behavior for the students who did not pass on their first attempt. Since the majority of traces in this cluster passed on the first short attempt, it is likely that a significant fraction of traces in this cluster resulted from students obtaining the answer from another source, rather than making a lucky guess. Out of at least 815 total traces in this causal net, there were at least 597 traces of passing after the first short attempt, 98 from the second short attempt, and 63 from the third short attempt. Students were not able to pass after making a failed fourth or fifth short attempt (at least 57 traces). Figure 4C outlines this causal net.

##### Causal Net 3: Normal, Then Short Attempts

**Figure 5:**
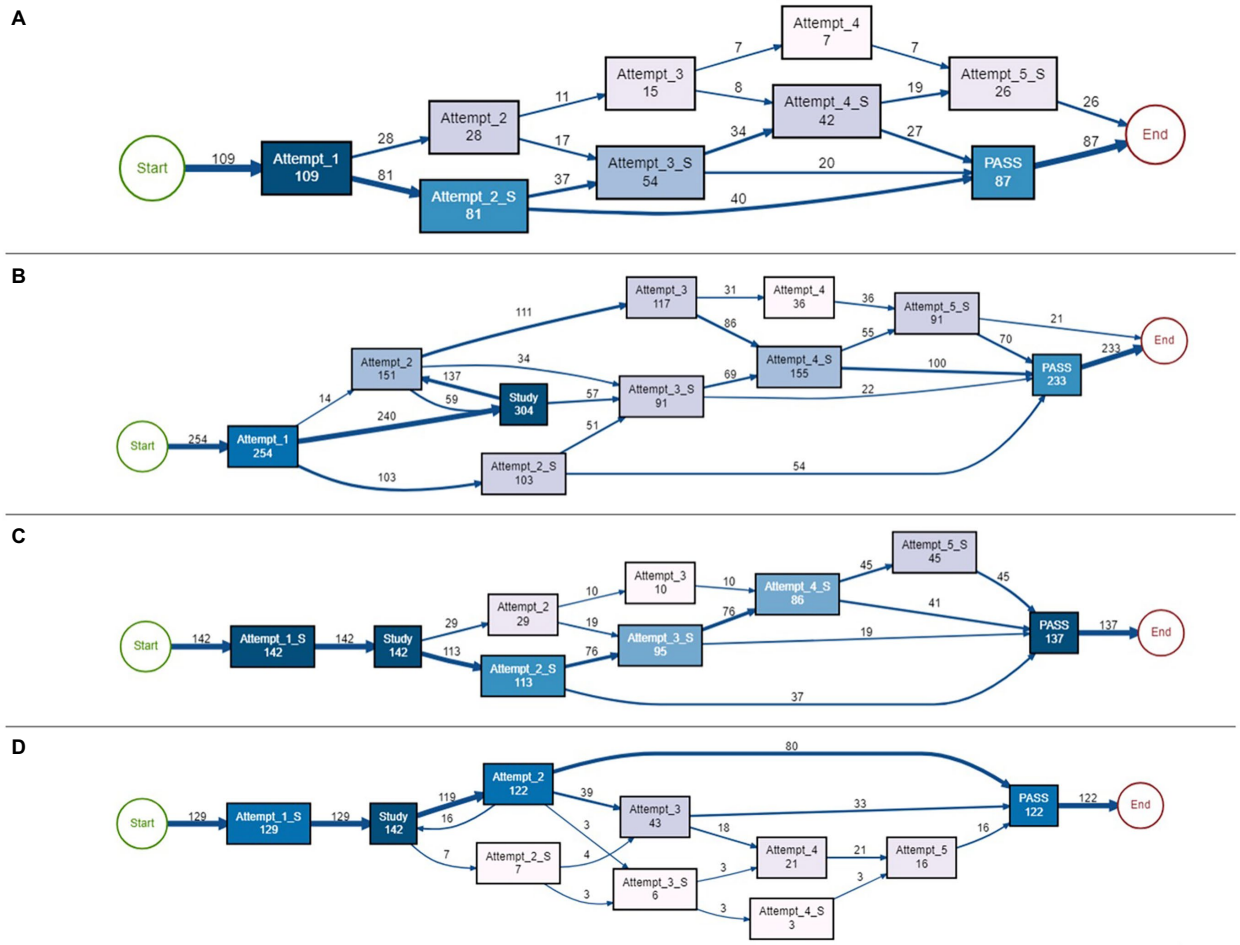
Causal nets. **(A)** Normal, then short attempt, Causal net 3; **(B)** attempt, study, many short attempts, Causal net 5; **(C)** short attempt, study, many short attempts, Causal net 6; **(D)** short attempt, study, attempt, pass, and Causal net 7.

This causal net demonstrates traces of behaviors with many short attempts after making at least one normal attempt. All students in this cluster (at least 109 traces) began completing the module by making a longer first attempt. Then, some students (at least 28 traces) made a long second attempt, with at least 11 traces followed by a normal third attempt, and at least seven traces followed that by a normal fourth attempt. However, none of these normal attempts led to passing the quiz. In this cluster, the only attempts that did lead to passing the quiz were short attempts. These short attempts were either made after the first failed attempt (at least 81 traces) or after three (at least 20 traces) or four (at least 27 traces) short attempts. There were at least 26 traces of failed fifth attempts as well. It is interesting to note that in this cluster, students did not make any study attempts, regardless of the attempt being normal or short. From an SRL perspective, these students seem to be demonstrating some planning or even monitoring behaviors, and the adaptations they were making were to shift from making normal attempts to short attempts to pass the assessment. Perhaps these students generated low self-efficacy in their ability to pass the assessment after their first failed attempt, and therefore did not feel exerting a substantial amount of effort would help them anyways, leading to more guessing-type behaviors. As such, perhaps these students were demonstrating self-regulation by making plans and adaptations to those plans; however, these might not have been the most desirable self-regulatory strategies needed to master the course material. Out of the at least 109 traces included in this cluster, at least 87 traces led to passing the assessment and at least 26 traces led to ending the module without passing. See Figure 5A for the overview of this causal net.

##### Causal Net 5: Attempt, Study, Multiple Short Attempts

This causal net can be categorized by students making both normal and short attempts after making study attempts. Out of at least 254 traces in this cluster, no one passed after the first attempt, which was a normal attempt. After this failed first attempt, most students (at least 240 traces) studied the material. Interestingly, traces following studying or making a normal second attempt did not lead to passing the module but making a short second attempt (at least 54 traces) did lead to passing. In fact, the only attempts that did lead to passing were short attempts, which occurred right after studying or after making more attempts (at least 22, 100, or 70 traces of passing after a third, fourth, or fifth short attempt, respectively). Making long attempts never led to passing the module. From an SRL perspective, it seems these students were monitoring their performance and were making adaptations (e.g., switching from normal to short attempts), but it seems like these students were not able to make accurate judgments of their understanding, demonstrated by making several attempts before passing the module, leading to changing to more guessing. Some students seemed to want to master the material, demonstrated by studying, but some students did not study at all, meaning they focused on their performance from the beginning. There were at least 233 traces of passing and only 21 traces of ending after a failed fifth attempt, so these students seemed persistent, and demonstrated self-regulation by adapting, but then possibly gave up and guessed until passing the module. Figure 5B demonstrates the traces in this cluster.

##### Causal Net 6: Short Attempt, Study, Multiple Short Attempts

This cluster net has similar characteristics as the above (Figure 5B), however in this cluster, all students (at least 142 traces) began with a first failed short attempt followed by studying the content. Only short attempts led to passing the module after two (at least 37 traces), three (at least 19 traces), four (at least 41 traces), or five (at least 45 traces) attempts. In this cluster, all students passed the assessment (at least 137 traces). From an SRL perspective, these students appear to be strategic planners. It is possible they quickly evaluated having low prior knowledge and therefore made a short attempt so they could proceed to studying the material they knew they needed to learn. After studying, most students judged their understanding of the material. Some students were accurate and passed the assessment. However, the majority made a third or fourth attempt before passing, demonstrating their content mastery was still not perfect, but instead of going back to studying, they continued making short attempts. Perhaps these students started with the strategy to master the content, but after being unsuccessful, most of them adapted to trying to pass the assessment with minimal effort, like previous clusters. The causal net can be seen in Figure 5C.

##### Causal Net 7: Short Attempt, Study, Attempt, Pass

This causal net is similar to the one seen in Figure 5C where all students started with a short failed first attempt followed by studying. However, what differentiates this one is that students only passed after making two (at least 80 traces), three (at least 33 traces), or five (at least 16) normal attempts. In addition, this is the only cluster that has some traces of students returning to the instructional materials with an earlier study event (at least 16 traces). The majority of traces in this cluster involve passing after making a second normal attempt, but all students did pass the module. From an SRL perspective, this suggests these students did assess needing to study the material and therefore made a short first attempt to get to the content quickly, and even if not successfully passing after the next attempt, students did spend time reading the question, suggesting they were monitoring their understanding of the question before answering. Even if they still did not pass, they did not give up and resort to guessing, at least before spending more time reading the question. For the few traces of short attempts, perhaps these students did try to guess, but adapted this strategy to spend more time reading the question to ensure they answered it correctly. In comparison to other causal nets, this cluster did not demonstrate successful quick guessing behaviors. See Figure 5D for the representation of this causal net.

In the remainder of this paper, we will refer to the seven module-level clusters as Causal Nets 1–7, to better distinguish from sequence-level clusters discussed below.

#### Sequence-Level Clusters

After outlining the seven causal nets, we wanted to determine whether students engaged in these behaviors repeatedly and consistently throughout the OLM sequence or were only adopting certain strategies occasionally. We investigated this by hierarchical agglomeration clustering at the sequence level (i.e., sequence-level clusters).

**Figure 6:**
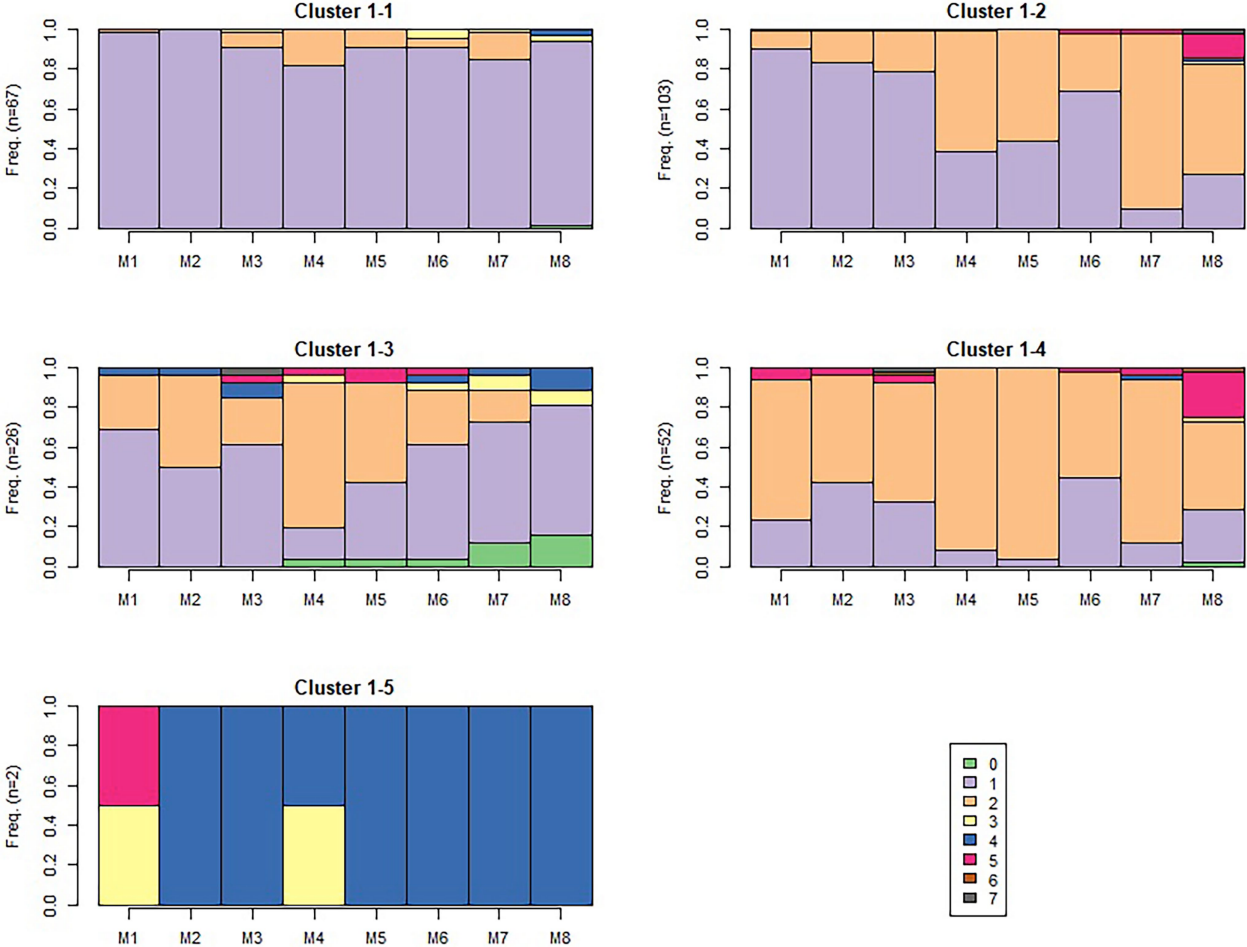
Sequence 1 sequence-level clusters. The number of students in each s-cluster is shown on the left of each figure.

**Figure 7:**
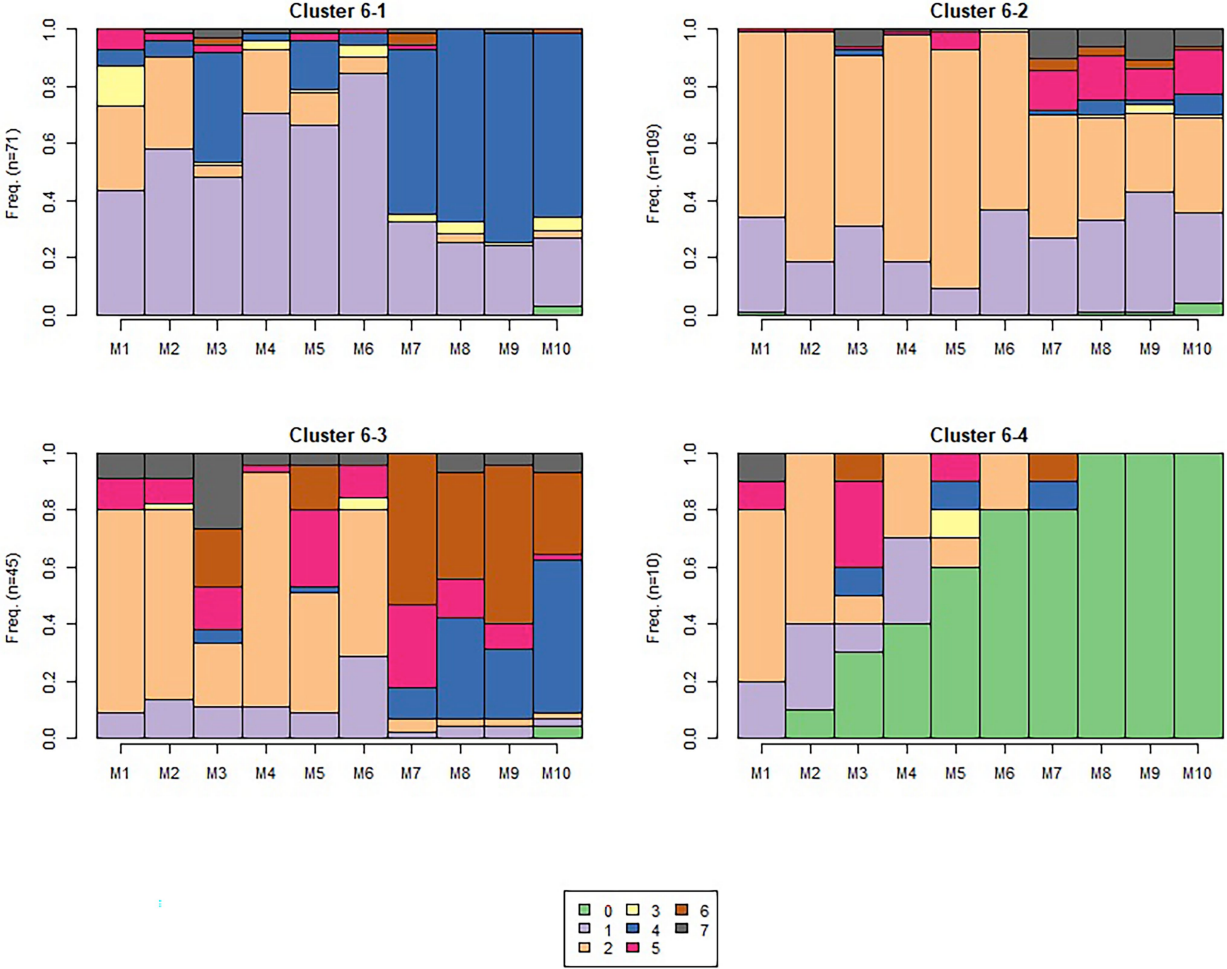
Sequence 6 sequence-level clusters. The number of students in each s-cluster is shown on the left of each figure.

**Figure 8:**
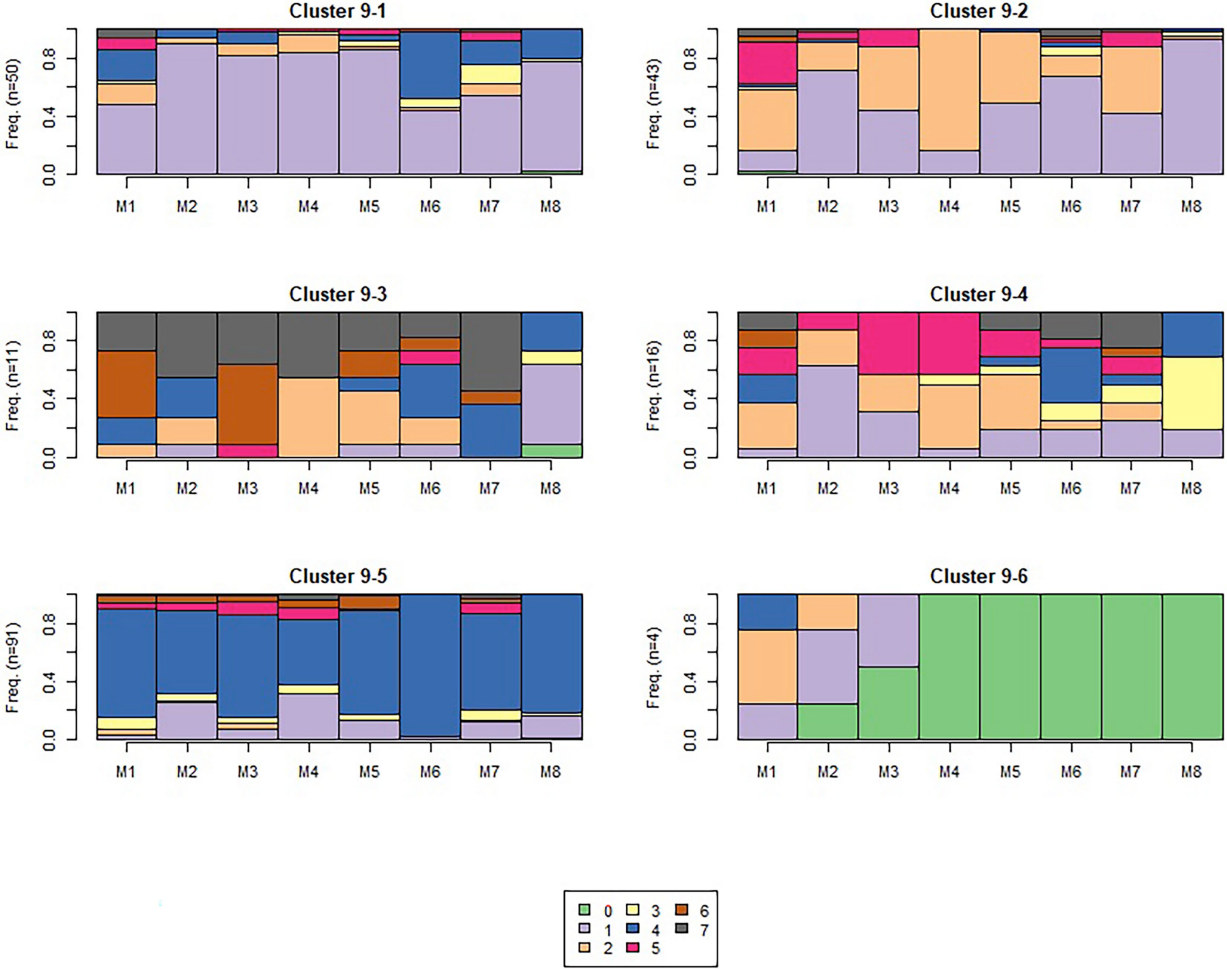
Sequence 9 sequence-level clusters. The number of students in each s-cluster is shown on the left of each figure.

The algorithm detected 5, 4, and 6 sequence-level clusters for sequences 1, 6, and 9 respectively, as visualized in Figures 6–8, which show the frequency of observing different module-level clusters (or causal nets) for each OLM using stacked bar charts, with the height of each bar representing the fraction that a given module-level cluster was observed for a given OLM. In all three figures, causal net 0 is used to indicate that the student did not interact with the given module. Based on the s-clusters, we determined that students did demonstrate a shift from engaging in behaviors for some modules at the beginning of the semester, to different behaviors 7 and 15 weeks into the semester. We will describe the dominant sequence-level clusters for each sequence below.

##### Sequence 1

In the beginning of the semester, we see the majority (but not all) of the traces in the s-clusters contained causal nets 1 and 2. This indicates that most students made normal attempts, and/or also studied the material upon failing their first attempts. Sequence-level cluster 1-1 (see Figure 6) is dominated by m-cluster 1 (*normal attempts and passing*); however, there is more observation of causal net 2 (*attempt, study, attempt, pass*) in sequence-level clusters 1-2, 1-3, and 1-4, suggesting many students did engage in study behaviors at the beginning of the semester. Sequence-level cluster 1-5 predominantly contains causal net 4 (*short attempt and pass*), likely a guessing-type behavioral cluster. It is important to make note of this cluster because while it only had two students at the beginning of the semester, similar behavior patterns will become more dominant toward the end of the semester, as detailed later. In general, evidence from these clusters demonstrates the majority of students were engaging in effective self-regulatory processes at the beginning of the semester.

##### Sequence 6

In the middle of the semester (7 weeks into the semester), we still observed some traces of causal nets 1 and 2; however, there appears to be more traces of the other m-clusters (see Figure 7). Sequence-level cluster 6-1 has many traces of causal net 1 (*normal first or second pass*) with many traces of causal net 4 (*short attempt and pass*) as well, and with some, but fewer traces of causal net 2 (*attempt, study, attempt, pass*) and 3 (*normal, then short attempt*). Sequence-level cluster 6-2 still reveals traces of causal nets 1 and 2, but with some causal net 5 too (*attempt, study, multiple short attempts*). Sequence-level cluster 6-3 demonstrates many more traces of causal nets 4, 5, 6, and 7 (all m-clusters with short attempts), with fewer traces of causal nets 1 and 2. Although s-cluster 6-4 has traces of several causal nets, it has the most traces of causal net 0, which was used to indicate the student did not interact with the module. This is likely due to students dropping the course prior to the add/drop period. Overall, from what we see in these sequence-level clusters, as we monitor traces of student behaviors across the semester, we see a transition to engaging in what seems like some effective self-regulatory behaviors, but also students are starting to engage in more guessing-type behaviors.

##### Sequence 9

Sequence 9 was administered toward the end of the semester (see Figure 8). From these sequence-level clusters, we again see the transition from engaging in more of causal nets 1 and 2 in the beginning of the semester to a shift to other causal nets that include short attempts and fewer study behaviors. This is especially apparent in more traces with a high frequency of causal net 4 (*short attempt and pass*). We do still see some traces of causal nets 1 and 2 (indicative of more effective self-regulatory behaviors), seen in sequence-level cluster 9-1 and sequence-level cluster 9-2. However, sequence-level clusters 9-3 and 9-4 seem to have a broad range of causal nets—specifically, 4, 5, and 6. These clusters all include short attempts, perhaps indicative of students engaging in a combination of study and guessing behaviors to finish the modules. Sequence-level cluster 9-5 is dominated by m-cluster 4 (*short attempt and pass*), which is the most indicative of guessing behaviors. Sequence-level cluster 9-6 is dominated by traces of not completing the modules (causal net 0, see above). In comparison to the previous sequences, these traces suggest students were not engaging in effective learning behaviors and were guessing to complete the modules. It is important to note that this behavior was not as prevalent across s-clusters for the two earlier sequences in the semester. This can be indicative that by the end of the semester, students have accumulated a sufficient amount of course credit, and were aiming to ensure they were passing their courses with acceptable scores, but also reserving effort—in alignment with a performance-oriented goal orientation (Elliot and Murayama, 2008).

### Research Question 2: To What Extent Do Students’ SRL Behaviors and AGQ Responses Change Over the Semester?

#### Change in SRL Behaviors

To describe a student’s shift in SRL strategy, we first sorted the sequence-level clusters into five different types, according to the frequency and type of causal nets observed within that sequence-level cluster. We then assigned a score (*S*) to each type, as listed in Table 1. Table 1 outlines which SRL strategy type scores are represented in each sequence-level cluster for each sequence 1, 6, and 9. For example, SRL strategy pass or study (SRL strategy type score 2) can be found in sequence clusters 2, 3, and 4 in sequence 1, sequence cluster 2 in sequence 6, and sequence cluster 2 in sequence 9. SRL strategy varied strategy (SRL strategy type score 3) cannot be found in any sequence clusters in sequence 1, however it can be found in sequence cluster 3 for sequence 6 and sequence clusters 3 and 4 for sequence 9. In Table 1, lower score numbers correspond to interactions that closely mirrored effective or desirable course interactions, such as learners passing on the first attempt or studying after the first failed attempt. Higher score numbers correspond to less desirable interactions, such as guessing. We then further described transitions between neighboring SRL strategies (Δ*S* = 1) as “Moderate” transitions and those between more distant strategies (Δ*S* > 1) as “Large” transitions. For example, if a student belongs to sequence-level cluster 1-1 (Type score of 1) and 6-3 (Type score of 3), we consider this a “Large” transition. The relative frequencies of same, moderate, and large transitions are listed in Table 2.

**Table 1:** S-Clusters sorted by dominant SRL strategy type score across the semester.

SRL strategy type score (*S*)	Sequence (S)–S-Cluster (SC)		
	Sequence 1	Sequence 6	Sequence 9
Initial pass (1)	S1-SC1	S6-SC1	S9-SC1
Pass or study (2)	S1-SC2, S1-SC3, S1-SC4	S6-SC2	S9-SC2
Varied strategy (3)	–	S6-SC3	S9-SC3, S9-SC4
Short pass (4)	S1-SC5	–	S9-SC5
Abort (5)	–	S6-SC4	S9-SC6

S, sequence; SC, sequence cluster.

**Table 2:** Frequencies of self-regulated learning (SRL) transitions between course module sequences.

Sequence transition	Large	Moderate	Same	Total
1–6	6.4%	45.1%	48.5%	235
6–9	32.1%	39.1%	28.8%	212

#### Change in AGQ Responses

Based on students’ RCI scores (see section “AGQ Change Scores”, above) we categorized scores according to Jacobson and Truax (1991) who stated RCI scores beyond |1.96| are statistically unlikely (*p* < 0.05) without the occurrence of real change between the set of test scores in question. Therefore, we define a decrease in goal endorsement as an RCI of −1.96 or less and an increase in goal endorsement as an RCI of 1.96 or larger for any of the four goal orientation profiles (Jacobson and Truax, 1991; Fryer and Elliot, 2007). RCI scores that fell within |1.96| were classified as a non-significant change, as was done in Jacobson and Truax (1991) and Fryer and Elliot (2007). See Table 3 for the breakdown of scores by goal orientation profile.

**Table 3:** Reliable change index (RCI) change % [*n*] from AGQ-R 1 to AGQ-R 2.

	Decrease	Increase	Non-significant change	Total
MAP	5.9% [14]	2.1% [5]	92% [219]	238
MAV	0.4% [1]	4.2% [10]	95.4% [227]	238
PAP	3.8% [9]	2.5% [6]	93.5% [223]	238
PAV	3.8% [9]	3.4% [8]	92.9% [221]	238

MAP, mastery approach; MAV, mastery avoidance; PAP, performance approach; PAV, performance avoidance.

#### Change in SRL in Relation to Change in AGQ Responses

A Kruskal-Wallis test found a significant relationship between students’ performance approach RCI scores (RCI_PAP) and end of term (from seq6 to seq9) behavior transition sequence-level cluster membership, *KW*(2, *n* = 205) = 6.275, *p* = 0.035. Students who stayed the same in their course behaviors between midterm and end of term (i.e., did not change SRL behaviors, based on shifts in students’ sequence-level cluster membership from seq6 to seq9) had larger changes in their performance approach scores (*M*_Rank_ = 118.86) than students who made moderate shifts in behavior (*M*_Rank_ = 93.34). Results were not significant for the other RCI scores (MAP, MAV, and PAV).

### Research Question 3: How Do Changes in Observed SRL Behavior and AGQ Responses Relate to Students’ Learning Outcome?

For this research question, we sought to compare change in SRL and AGQ with course exam scores by comparing exam scores between sequence clusters and correlating exam scores with AGQ change scores.

#### SRL Behavior Changes and Exam Scores

**Figure 9:**
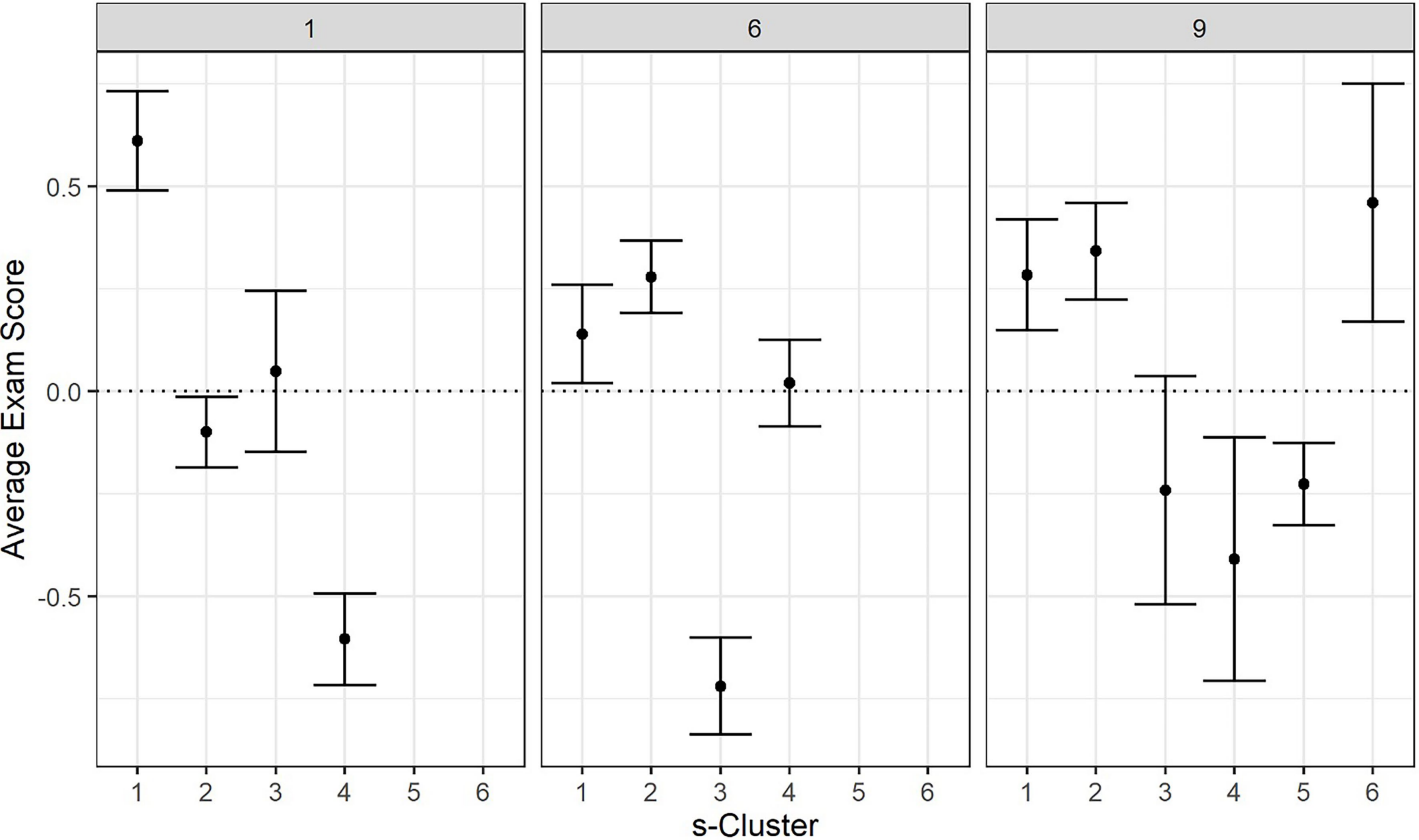
Average exam scores by sequence-level clusters 1 (left), 6 (middle), and 9 (right).

Within each of the three OLM sequences, ANOVA tests reveal that the exam scores between different s-clusters were significantly different (see Table 4). *Post-hoc* pairwise comparison using Tukey HSD tests revealed a total of seven pairs of s-clusters that had significantly different exam scores, as listed in Table 5 [*p*-values were adjusted using the fdr method (Benjamini and Hochberg, 1995)]. In each pair, s-clusters classified as either “initial pass” or “pass or study” had higher exam scores than other types of s-clusters. The only exception is sequence-level cluster 1-4, which is classified as “pass or study,” yet had significantly lower exam scores compared to sequence-level clusters 1-1 and 1-2. Sequence-level cluster 1-4 had a higher fraction of study events (causal net 2) than sequence-level clusters 1-1 and 1-2, especially on the first two modules. See Figure 9 for a breakdown of exam score for each s-cluster at sequences 1, 6, and 9.

**Table 4:** ANOVA results for exam scores between sequence-level clusters.

Sequence	*F*-Statistic	*p*-Value	Partial Eta Squared
1	*F*_4,230_ = 13.3	<0.001	0.187
6	*F*_3,224_ = 13.2	<0.001	0.150
9	*F*_5,209_ = 4.07	0.00152	0.089

**Table 5:** *Post-hoc* comparisons for exam scores between s-clusters.

Sequence	Sequence-level cluster comparison	Estimated difference
1	1:2^***^	0.71
1	1:4^***^	1.21
1	2:4^*^	0.50
6	1:3^***^	0.86
6	2:3^***^	1.00
9	1:5^*^	0.51
9	2:5^*^	0.57

* *p* < 0.05;

*** *p* < 0.001.

#### AGQ Response Changes and Exam Scores

A Pearson correlation did not find a statistically significant correlation between students’ mastery approach AGQ change score and their final exam score [*r*(227) = 0.044, *p* = 0.505], their mastery avoidance AGQ change score and their final exam score [*r*(227) = 0.067, *p* = 0.317], their performance approach AGQ change score and their final exam score [*r*(227) = −0.006, *p* = 0.926], or their performance avoidance AGQ change score and their final exam score [*r*(227) = 0.114, *p* = 0.086].

## Discussion

The goal of this study was to examine how students engaged in self-regulatory actions during physics learning with OLMs, and whether their self-regulatory behaviors changed throughout the semester. We also sought to examine students’ self-reported goal orientations and whether those changed over time (i.e., throughout the semester) as well. Finally, we assessed the relationship between SRL and AGQ with exam scores for the course. In the next section, we discuss our overall findings from our research questions, followed by a discussion of the implications of these findings.

### Research Question 1: What Are the Different Types of SRL Processes Students Employ in a Self-Paced Online Learning Environment?

In general, evidence from the seven causal nets demonstrates students took different approaches to trying to complete the assignment, yet similar behavior patterns were found across groups of students. For example, some students took the time to make their first assessment attempt, but others made short attempts, possibly because they were either guessing, had obtained the answer from another source, or wanted to get to reading the study materials right away. Although all demonstrating self-regulation, these behaviors suggest students set different achievement goals within the learning modules. It seems some students engaged with provided learning materials as a means of mastering the content, while others wanted to ensure they could pass the assessment components as quickly, or with as little effort, as possible. It is important to note we cannot confirm each students’ established goals, but we believe examining how learners engaged in these modules over time can demonstrate whether these behaviors are spontaneous for one module or are more representative of a student’s typical behaviors across the semester. We addressed this by examining sequences of engaging in these m-clusters.

Results from sequence analysis demonstrated a shift in the frequency of various m-clusters among the student population, indicative of a shift in students’ SRL behaviors over time. More specifically, some students appeared to shift from more effective to less effective SRL strategies, suggesting they no longer seemed to set the goal of mastering the content. Rather, it seems they were focusing more on their grades and performance in the class. This was especially apparent by the shift in the number of students adopting causal net 4 (short attempt and pass), which was very low in the first sequence of the course but became much higher in sequences 6 and 9 toward the end. By the end of the semester, students often feel overwhelmed with the amount of work they need to complete to pass their courses, requiring motivation regulation (Schunk and Zimmerman, 2012). Therefore, it is possible that we are seeing this shift because students are realizing the work they need to complete not only in this class, but also all of their other courses as well. This does not imply they no longer value the mastery of course content, but rather the letter grade calculation takes priority in this case. As introductory physics is a challenging course, this might have been even more apparent to these students. Future work is needed to confirm what is causing these shifts in student behavior traces throughout the semester.

### Research Question 2: To What Extent Do Students’ SRL Behaviors and AGQ Responses Change Over the Semester?

When examining change in SRL behaviors, students did not make large shifts from sequence 1 to sequence 6 (6.4%), especially in comparison to making a moderate shift (45.1%) or making no shift (48.5%). However, this changed from sequence 6 to sequence 9 where we see a much larger percentage of large shifts (32.1%), and still moderate (39.1%) or no shifts (28.8%); however, to a lesser extent than the previous sequence shift. In addition, the results demonstrated that students’ SRL strategy can shift abruptly on a shorter timescale, such as a sudden shift in strategy at the middle of sequence 6.

Those strategy shifts may have been caused by students’ sense of urgency to increase their course grade. It could be that students suddenly became aware of the fact that they were not achieving a desirable grade in the course and came to conclude that they were spending too much time reading the content with little improvement on assessment performance. Another interpretation could be related to students’ reaction to changes in content difficulty. Since the modules in general get progressively more difficult over time, students were having a more difficult time completing the later modules successfully, which eventually lead to more guessing behaviors. Therefore, similar to our earlier interpretation of behaviors for research question 1, it is possible students are making a shift later on in the semester because they are suddenly focusing on ensuring they earn an acceptable grade in the course, so instead of focusing their attention on mastering the content, they are ensuring they are performing well.

For AGQ responses, although some students demonstrated a shift in their responses, it was much less than expected. Specifically, scores for mastery approach changed the most, yet it was only for a 5.9% increase and 2.1% decrease (and 92% no significant change). In general, between 1 and 10 students along different dimensions shifted their responses to the AGQ after completing the questionnaire a second time, which demonstrates *some* students do change their self-reported goal orientation, despite the majority of students being consistent in their response.

It is interesting that students who did not demonstrate a shift in SRL behaviors (i.e., remained in the same cluster from sequence 6 to sequence 9) demonstrated larger changes in performance approach scores compared to students who demonstrated a moderate shift in SRL behavior from sequence 6 to sequence 9. Perhaps these students were better able to re-align their goal orientation with their SRL behaviors at the end of the semester. In other words, their SRL behaviors did not change from sequence 6 to 9, but their reported goal orientation did. Based on this result, now that we know there is a relationship between students who are not changing their SRL behaviors, but are changing their AGQ responses, future work is needed to examine *why* they are making these changes or not.

### Research Question 3: How Do Changes in Observed SRL Behavior and AGQ Responses Relate to Students’ Learning Outcome?

Findings from this research question outlined that not only were we able to outline the changes in SRL and AGQ behaviors throughout the semester, changes in SRL behaviors were also associated with performance in the course. As expected, in most cases students who engaged in m causal nets 1 and 2 had higher exam scores compared to their peers. However, sequence-level cluster 1–4, which did contain predominantly causal nets 1 and 2, is an exception for having significantly lower exam scores. It is possible that even though students seemed to be engaging in effective SRL behaviors, this does not always guarantee greater performance (Taub and Azevedo, 2018, 2019). For example, if a student is focusing on mastering the content, perhaps they are not focusing on content that is included in the test. The student, therefore, has high procedural knowledge of engaging in SRL strategies, but might not have high levels of content knowledge (Azevedo and Taub, 2020). Since sequence-level cluster 1–4 contains more causal net 2, it could also be that those students had less incoming knowledge compared to their peers. It would be interesting to investigate students’ levels of procedural and conditional knowledge in addition to their content knowledge, as well as their content knowledge prior to instruction.

In addition, findings did not reveal significant correlations between course scores and change in response scores to the AGQ, likely due to the low frequency of students who completed the AGQ at the end of the semester, resulting in an incomplete picture of how students’ goal orientation shifted by the end of the course. As such, future work should seek to encourage the completion of the AGQ for all participants at more timepoints throughout the semester.

However, there were significant relationships between AGQ scores taken at different points in the semester (i.e., when using raw scores instead of change scores), which exceeds the scope of this paper given that our research question sought to examine change in both SRL behaviors and AGQ scores, not raw data. However, it is worth noting that perhaps the significance of the relationship between exam score and raw AGQ scores suggests students are focusing on achieving both mastery and performance at the beginning of the semester (approach), followed by a shift to focusing on avoiding failure in mastering the content or performing poorly (avoidance). Put differently, there is a potential shift from *approach to avoidance* raw scores being significant. This aligns with other findings in that students are demonstrating a shift in behavior from the beginning to middle to end of the semester, and future research examining the nature of this change will be important.

### Limitations and Future Directions

It is important to acknowledge that although our research yielded interesting and informative results, we must address the limitations from our study as well. First, although we administered the AGQ-R three times at the beginning, middle, and end of the semesters, there were much fewer responses to the third AGQ administration (*n* = 40) and we were therefore unable to include it in our analyses. In addition, we only performed clustering on sequences 1, 6, and 9 of the semester. In future studies, we will expand our analyses to include all sequences from the course and find methods to improve survey response rate toward the end of the semester. In addition, our method for producing the Gower dissimilarity matrix was not exhaustive—we simply chose between several different weights selected to emphasize certain phases of SRL. In future studies, we plan to use bootstrapping methods to more comprehensively search the space of Gower weights to find the weights for which the cluster membership most closely represents the underlying structure of the data.

Regarding the multilevel clustering analysis scheme, one outstanding limitation is that the current analysis simplified students’ interactions with the instructional materials into a single binary variable. Future analysis should incorporate more interaction details, such as the number of practice questions answered or time spent on the materials, to better reflect students’ study strategies. A second technical limitation is that the weights of the Gower dissimilarity coefficients were chosen so that it produced well-structured clustering structures for less than 10 clusters. Future studies should explore whether there are other sets of parameters that result in well-structured clusters, which could emphasize a different aspect of the SRL process, such as content knowledge mastery and problem-solving ability.

Our results left a lot of room for interpretation. We used a theoretical framework and based our findings on the information processing theory of SRL; however, these are speculations. In other words, we know which actions students completed, but we do not know *why* they performed these actions. Therefore, in future studies, we will seek to explore this question and investigate why students are changing their behaviors or motivations throughout the semester. Possible studies can include incorporating prompts to foster student reflections throughout the semester. In addition, measuring student achievement goals is not the only factor at play here. Thus, we can administer additional questionnaires to complement the AGQ-R to gauge student motivation (e.g., self-efficacy and task value), emotions (e.g., emotions and values and emotion regulation), and students’ perceived use of self-regulatory processes. It might also be helpful to conduct student interviews that ask them to discuss the processes they use while engaging in the learning modules in the course.

These potential future directions pave the way toward developing online learning modules or MOOCs that provide adaptive support based on student behaviors. For example, if the system detects many student-level traces of causal net 4 (i.e., guessing), the system can suggest the student spend more time reading through questions or spending time studying the course material. This can help to ensure all students are successfully learning course materials while also earning acceptable grades to help them pass their courses.

## Conclusion

This paper examined college students’ SRL behaviors and self-reported AGQ as they completed one semester of college-level introductory physics during the Fall 2020 semester, using OLMs as homework and self-study materials. Based on our findings, we propose it is informative for the study of SRL to examine the changing nature of SRL and AGQ because we did find evidence for that in our results. Our results therefore confirm what is posited in the information processing theory of SRL (Winne and Hadwin, 1998, 2008; Winne, 2018)—that SRL should be viewed as a series of events that unfold during learning. Our results have useful implications for designing future online and blended courses because we are progressing toward fostering the use of effective SRL throughout the entire semester. In future studies, it would be helpful to determine actions at the student-level, which could be used to inform the design of future OLMs or MOOCs that provide adaptive feedback based on individual student behavior. We conclude that there is still significant work ahead in investigating and fostering SRL during online and blended learning settings, but this paper provides a good blueprint for the types of analyses helpful for investigating how students’ learning strategies as well as goals and orientations change over the semester.

## Data Availability Statement

The raw data supporting the conclusions of this article will be made available by the authors, without undue reservation.

## Ethics Statement

The studies involving human participants were reviewed and approved by University of Central Florida Institutional Review Board (STUDY00000994). Written informed consent for participation was not required for this study in accordance with the national legislation and the institutional requirements.

## Author Contributions

All authors contributed to the conception of the work and revised the final manuscript. MT led the conceptualization and writing of the paper. ZC designed and conducted the study and proposed the analysis scheme. ZC, AB, and TZ conducted the statistical analyses. MT, AB, TZ, and ZC all contributed sections to the manuscript. All authors also provided several rounds of edits on the manuscript.

## Funding

This research is partly supported by the National Science Foundation (NSF), Grant No. DUE-1845436. Any opinions, findings, conclusions, or recommendations expressed in this material are those of the author(s) and do not necessarily reflect the views of the National Science Foundation.

## Conflict of Interest

The authors declare that the research was conducted in the absence of any commercial or financial relationships that could be construed as a potential conflict of interest.

## Publisher’s Note

All claims expressed in this article are solely those of the authors and do not necessarily represent those of their affiliated organizations, or those of the publisher, the editors and the reviewers. Any product that may be evaluated in this article, or claim that may be made by its manufacturer, is not guaranteed or endorsed by the publisher.

## Footnotes

^1^ https://www.aaas-iuse.org/resource/course-design/
